# Presence of glyphosate in endometrial cancer tissue: A cross-sectional study

**DOI:** 10.1016/j.toxrep.2026.102255

**Published:** 2026-04-23

**Authors:** Márkó Unicsovics, Apolka Szentirmay, Györgyi Fekécs, Patrik Plank, Zsófia Molnár, György Nagyéri, Katalin Posta, Nándor Ács, Szabolcs Várbíró, Levente Sára, Zsuzsanna Szőke

**Affiliations:** a Department of Obstetrics and Gynecology, Semmelweis University, Baross utca 27, Budapest 1088, Hungary; b Department of Animal Biotechnology, Agribiotechnology and Precision Breeding for Food Security National Laboratory, Institute of Genetics and Biotechnology, Hungarian University of Agriculture and Life Sciences, Szent-Györgyi Albert utca 4, Gödöllő 2100, Hungary; c Department of Neurobiology, Institute of Biology, Faculty of Science, University of Pécs, Ifjúság útja 6, Pécs 7624, Hungary; d Department of Microbiology and Applied Biotechnology, Institute of Genetics and Biotechnology, Hungarian University of Agriculture and Life Sciences, Szent-Györgyi Albert utca 4, Gödöllő 2100, Hungary; e Department of Obstetrics and Gynecology, University of Szeged, Semmelweis utca 1, Szeged 6725, Hungary

**Keywords:** endometrial cancer, glyphosate, mycotoxins, zearalenone, endometrial tissue

## Abstract

Glyphosate (GLY), a widely used herbicide classified as a probable human carcinogen, has an unclear role in hormone-dependent malignancies. This cross-sectional study investigated the presence and elevated concentration of this compound in endometrial tissues and its potential link to uterine cancers, also exploring interactions with other natural environmental contaminants such as mycotoxin Zearalenone (ZEN). Patients with endometrial cancers and age- and Body Mass Index-matched controls with benign endometrial diseases were recruited. Serum and excised endometrial tissue samples were analyzed for GLY, ZEN, and related physiological-hormonal indicators. Results revealed significantly elevated serum GLY levels in patients with high-grade endometrial cancer compared to controls (p < 0.05). Similarly, endometrial tissues from cancer patients, particularly those with high-grade malignancy, exhibited higher GLY levels than controls. Notably, GLY concentrations were significantly greater in endometrial tissue than in serum across all participant groups. No significant correlations were found between hormonal/physiological indicators and GLY levels. A strong positive correlation was observed between endometrial GLY and ZEN levels. This study is among the first to detect the presence and elevated concentration of GLY in human endometrial tissue, raising concerns that GLY exposure may be a significant factor in hormone-dependent diseases, particularly those mediated by estrogen or estrogen-like substances. The findings also highlight the need for further research into the endocrine-disrupting effects and long-term health consequences of GLY and various mycotoxins, as well as their potential synergistic involvement in the development of uterine cancers.

## Highlights

• Glyphosate was detected and quantified in human endometrial tissue for the first time.
• Endometrial glyphosate levels were significantly higher than serum levels.
• High-grade endometrial cancer was associated with elevated glyphosate concentrations in serum and tissue.
• Glyphosate levels strongly correlated with the estrogenic mycotoxin zearalenone, suggesting potential toxicological synergy.

## Graphical Abstract

**Figure:**
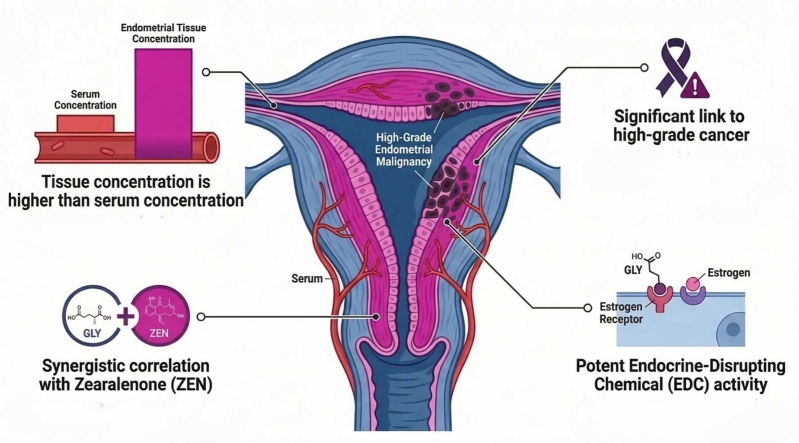

## 1 Introduction

Glyphosate (GLY) is a widely used non-selective herbicide [1]. Since the adoption of GLY-tolerant,often genetically modified crops, GLY-based herbicides (GBHs) have remained among the most common and extensively studied pesticides [2].

Due to their toxicity and residues, the applications of GBHs on tolerant crops can have both direct and indirect effects, including inter alia chelation, which may alter nutrient bioavailability and plant absorption [3]. Globally, concerns about the long-term health impacts of GLY and GBHs have increased; the World Health Organisation classified GLY as probably carcinogenic (Group 2B) in 2015 [4]. Human exposure through consumption of contaminated drinking water and food products such as tomatoes, paprika, seafood, tea, coffee, and honey has increased, with higher residue levels detected in some items [5], [6]. Evidence suggests a potential link between GLY and non-Hodgkin lymphoma, supported by animal studies associating it with various cancers. As endocrine-disrupting chemicals with estrogenic activity at low doses, GBHs may cause reproductive issues and interfere with endocrine functions by affecting aromatase and estrogen receptors [7], [8].

The uterus is particularly vulnerable to developmental disruptions caused by environmental pollutants such as mycotoxins, which can lead to decreased fertility and increase the risks of uterine cancer and fibroids [9], [10]. Uterine cancer (mainly endometrial cancer), is the sixth most common neoplasm worldwide and the most prevalent gynecological malignancy, with rising incidence over recent decades [11]. Its development is hormone-dependent, influenced by endogenous estrogen and progesterone levels, with risk factors including obesity, diabetes, and polycystic ovary syndrome that elevate estrogen levels [12]. Endometrioid endometrial cancer accounts for 80–90% of cases, often exhibiting high estrogen receptor 1 (ESR1) expression and estrogenic effects on the endometrium. Postnatal exposure to GBHs has been linked to endometrial hyperplasia in animal models, which may progress to cancer [13].

Numerous studies have established associations between GLY exposure and health issues, including various cancers. As a potential endocrine-disrupting chemical, GLY raises concerns among health advocates, who call for reevaluation of its safety at a national policy level. Despite these concerns, assessments by the European Commission and the European Food Safety Authority have concluded that GLY does not pose significant genotoxic or carcinogenic risks, leading to its reauthorization for use in Europe until 2033 [14].

Reviewing the current literature indicates potential carcinogenic effects of GLY, revealing overlapping biological pathways involved in endometrial cancer development and the estrogenic actions reported in in vitro and animal studies. However, clinical research directly linking GLY exposure to endometrial cancer remains limited. It is also important to recognize that multiple carcinogenic factors may cumulatively contribute to endometrial cancer development. Our previous studies have confirmed higher concentrations of mycotoxins such as aflatoxins and zearalenone (ZEN) in the endometrial tissue of affected patients [15]. ZEN and its metabolites can act as not only toxicogenic compounds but also xenoestrogens, binding to estrogen receptors and potentially promoting cancer progression. Furthermore, the synergistic effects of combined carcinogenic factors may accelerate tumor development, although data on the interactions among mycotoxins, endocrine disruptors such as GBHs, and GLY remain limited.

With our current study, we aimed to obtain a cross-sectional view of GLY level in endometrial tissue, compare it to serum GLY levels, and determine the possible association of higher level of GLY with uterine cancer formation. Additionally, we also wanted to consider and emphasize interactions of GLY with other relevant, acting endocrine disrupting chemicals/pollutants (EDCs), such as some mycotoxins.

## 2 Materials and methods

### 2.1 Patient recruitment and sample collection

Between September 8, 2023, and June 19, 2024, serum and endometrial tissue samples were obtained from patients. These patients were either undergoing treatment for previously histologically confirmed endometrial cancer or were reasonably suspected to have the condition. Additionally, samples were obtained from control patients who underwent treatment or surgery for non-malignant reasons during the same period of the study. All participants received comprehensive information, and sample collection was conducted after the approval of the regional ethics committee and signing of the relevant consent form. In all instances, samples were collected after fasting for at least 12 h.

Furthermore, data were collected on weight, age, obstetric history, comorbidities, medication use, smoking, and alcohol consumption. The patients' liver and kidney function laboratory values were evaluated, and endometrial thickness was measured via ultrasound examination. Patients with malabsorption, severe kidney or liver failure, thyroid disease, or significant weight changes in the recent period were excluded from the study.

Information on lifestyle habits related to the potential risk of environmental exposure, as reported by both the anamnesis and directly by patients, was also collected.

The concentrations of GLY, mycotoxins including total aflatoxins (Afs, B1, B2, G1, G2), deoxynivalenol (DON), fumonisin B1 (FB1), Ochratoxin A (OTA), ZEN, alpha-zearalenol (α-ZOL), T2/HT2 toxins, estradiol (E2), progesterone (P4), and estrone (E1) were quantified from serum samples following the methodology outlined below. All endometrial samples were subjected to histological evaluation. In instances of endometrial cancer, a mutation analysis, which is crucial for diagnosis, was also performed.

### 2.2 Measurements of GLY in Collected, Processed Samples

#### 2.2.1 GLY measurement from serum

A commercial kit (ABRAXIS Glyphosate Plate ELISA Kit, PN 500205, Gold Standard Diagnostics, Warminster, US) was used in this study. This kit provides a reliable and precise method for detecting GLY in serum samples. A manufacturer-validated procedure for serum extraction and measurement was adhered to. To prepare the samples for analysis, 500 µL of each crude serum sample was processed using Millipore Amicon centrifugal filter units. The samples underwent centrifugation at 8000 x g for 15 min to separate the supernatant from any solid particles or debris. After centrifugation, 300 µL of supernatant was transferred to another tube. To extract the GLY from the sample, 200 µL of ethyl acetate was added to the tube. The tube was vortexed for 30 s to ensure thorough mixing of the components. Following this, the tube was centrifuged again for 3 min at 8000 × g to separate the different phases in the sample. The aqueous phase at the bottom, which contained GLY, was carefully transferred to a new tube and further prepared for analysis using the kit components. Derivatization of the standards, controls, and prepared samples was conducted according to the manufacturer's instructions [16]. The cross-reactivity of the kit with glycine, AMPA, and glufosinate was negligible.

#### 2.2.2 GLY measurements from endometrial tissues

The measurements were performed using the commercial Kit (mentioned above). Although this kit is factory-validated primarily for human serum, its antibody system is specifically designed to handle and mitigate interfering factors present in complex biological matrices.

To adapt this kit for the analysis of endometrial tissue, methodological modifications were applied based on protocols published by Denžić et al. (2024), who successfully optimized GLY extraction from complex animal matrices, such as liver and adipose tissues. Because GLY is a highly polar molecule, an acidic aqueous extraction effectively releases it from the tissue. Subsequently, applying the ethyl acetate wash—a standard step in the serum protocol—removes lipid-soluble matrix contaminants. This combined approach ensures the kit receives the purified, aqueous medium for which it was designed.

### 2.3 Combined endometrium extraction and purification protocol

#### 2.3.1 Phase 1: tissue disruption and extraction

To release GLY from the cells into an aqueous buffer, 0.5 g of well-homogenized endometrial tissue was weighed into a centrifuge tube. Following the 1:5 ratio utilized by Denžić et al. (2024) for liver extractions, 2.5 ml of 1% formic acid in water was added as the extraction solvent. The mixture was vortexed with ceramic beads for 1 min, followed by an additional 15 min of vortexing, and then placed on a rotary shaker for 15 min to ensure complete extraction. For primary separation, the samples were centrifuged at 4000 rpm for 15 min at 4 °C to sediment coarse tissue debris. For filtration, 500 µL of the supernatant was pipetted into a Millipore Amicon Ultra 0.5 ml 10 kDa ultra-filter unit (if the sample was highly turbid, the volume was split between two filters). The units were centrifuged at 8000 x g for 15 min to remove proteins larger than 10 kDa that could interfere with antibody binding.

#### 2.3.2 Phase 2: chemical purification and lipid phase removal

To remove tissue lipids and other non-polar interfering substances, 300 µL of the clean, ultra-filtered supernatant was transferred to a new microcentrifuge tube. Next, 200 µL of ethyl acetate was added as an organic wash. The tube was vigorously vortexed for 30 s, followed by centrifugation at 8000 x g for 3 min to achieve phase separation.

#### 2.3.3 Phase 3: final recovery and ELISA measurement

The lower aqueous phase, containing the isolated glyphosate, was carefully aspirated and transferred to a new, labeled tube, while the upper ethyl acetate phase was discarded. Derivatization was then performed. If the tissue matrix prevented the collection of the full 250 µL aqueous phase, the manufacturer-approved half-volume protocol was employed: 125 µL of the sample was mixed with 500 µL of assay buffer and 50 µL of diluted derivatization reagent. The derivatized samples, standards, and controls were subsequently assayed and analyzed as directed.

### 2.4 Recovery assessment via tissue spiking

To evaluate the recovery of the adapted extraction method, blank (GLY-free) endometrial tissue homogenates (0.5 g) were spiked prior to extraction. A 2.5 ng/ml aqueous GLY working standard was utilized to ensure precise dosing and to mimic native tissue binding. Either 50 µL or 70 µL of this working solution was added directly to the 0.5 g blank tissue aliquots to achieve final tissue concentrations of 0.25 ppb (0.125 ng absolute amount) and 0.35 ppb (0.175 ng absolute amount), respectively. Crucially, the spiked tissues were incubated for 15 min prior to the addition of the 2.5 ml extraction solvent. This incubation step allowed the analyte to interact with the tissue matrix, ensuring the recovery experiments accurately reflected the extraction efficiency of endogenous tissue-bound GLY rather than merely the recovery of the standard from the solvent. Based on these repeated measurements, the recovery of GLY in the conducted assays was 79.31 ± 4.43%

### 2.5 Measurements of mycotoxins

Mycotoxin levels were quantified in endometrial tissue and serum samples from each group. The assays employed enzyme-linked immunosorbent assay (ELISA) method optimization for both serum and endometrial tissue samples, with ZEN, OTA, FB1, DON, T2/HT2 toxin, and Afs) quantified using immunoassays. For α-ZOL, gas chromatography-mass spectrometry (GC-MS) was utilized [15], [17]. As detailed in our previous publication [15], these mycotoxins (ZEN, α-ZOL, OTA, Afs, DON, FB1, and T2/HT2-toxins) were measured in serum and endometrial tissue samples using various techniques. ZEN was analyzed using an enzyme immunoassay kit with C18 column purification for serum and enzymatic (β-glucuronidase/aryl sulfatase) digestion, followed by methanol extraction for endometrial tissue. α-ZOL was measured by GC-MS following C18 column purification and methanol/acetonitrile reconstitution. OTA, Afs, DON, FB1, and T2/HT2 levels were determined using ELISA kits, with specific extraction and dilution procedures for both serum and endometrial tissue. All samples were measured in triplicate, and recovery rates were assessed for the endometrial tissue samples by spiking with determined concentrations of mycotoxins [15].

### 2.6 Measurements of hormones and other mediators

#### 2.6.1 Hormone analyses from serum

To quantitatively assess E1 levels in the serum, an ALPCO Estrone ELISA kit (Cat. No. 11-ESRHU-E01; Salem, US) was utilized. The immunoassay was performed according to the manufacturer's guidelines, with serum samples tested in triplicate. For the analysis of E2 and P4 in serum, NovaTec 17-beta-estradiol (Cat No: DNOV003, NovaTec Immundiagnostica, Dietzenbach, Germany) and NovaTec Progesterone (Cat No: DNOV006) were employed. These immunoassays were performed according to the manufacturer's instructions, and serum samples were measured in triplicate. The Atellica IM Thyroid Stimulating Hormone 3‑Ultra (TSH3‑UL) assay is for in vitro diagnostic use to quantitatively determine thyroid-stimulating hormone (TSH) levels in human serum using the Atellica Immunoassay Analyser 1600. (Siemens Healthineers, Cary, NC, USA).

#### 2.6.2 Clinical chemistry parameters

Standard, usual serum clinical chemistry parameters (AST, ALT, GGT and creatinine) were measured in routine processes using an Atellica Solution CH analyzer (Siemens Healthineers, Cary, NC, USA). The eGFR was determined using the Creatinine Equation for Glomerular Filtration Rate (CKD-EPI) equation, which incorporates the patient's serum creatinine level, age and sex.

Statistical analysis: Statistical analyses were performed using GraphPad Prism software (Version 10.1.1, 2023, Boston, MA, USA). To compare continuous variables, either an independent *t*-test or a Mann–Whitney test was utilized. Linear regression was applied to examine independent correlated factors, while Pearson correlation assessed the relationship between values. Multiple logistic regression was employed to predict the impact of various variables. A p-value of less than 0.05 was considered statistically significant.

Institutional Review Board Statement: The study was conducted according to the guidelines of the Declaration of Helsinki and approved by the Institutional Review Board of Semmelweis University. Reference No.: SE-RKEB 86/2023.

Informed Consent Statement: Informed consent was obtained from all subjects involved in the study.

## 3 Results

### 3.1 Investigated patient group

The study included 34 participants with a positive malignant histology; 11 were perimenopausal, and 23 were postmenopausal. Endometrial samples were collected from each in addition to serum. Endometrial intraepithelial neoplasia was established in four cases through histological investigation; six were high-grade endometrial cancer, and 24 were low-grade endometrioid endometrial cancer. Two patients were excluded from the low-grade group due to measurement inaccuracy. The 32 control patients were paired by their body mass index (BMI) and age. Serum and endometrial samples, as well as physiological, anamnestic, and lifestyle information, were collected from all control patients. In this group, 15 patients were perimenopausal, and 18 were postmenopausal. All included control patients had a benign histopathologic diagnosis. One had an endometrial polyp, two had simplex hyperplasia without atypia, and the remaining 30 had either atrophic endometrium or a negative diagnostic result. Only a part of the tissue samples of endometrial tissue were used for GLY and mycotoxin measurements throughout the endometrial sample process, since a sufficient amount of endometrial tissue needed to be left over for the required diagnostic, histopathologic, and staging procedures.

**Fig. 1:**
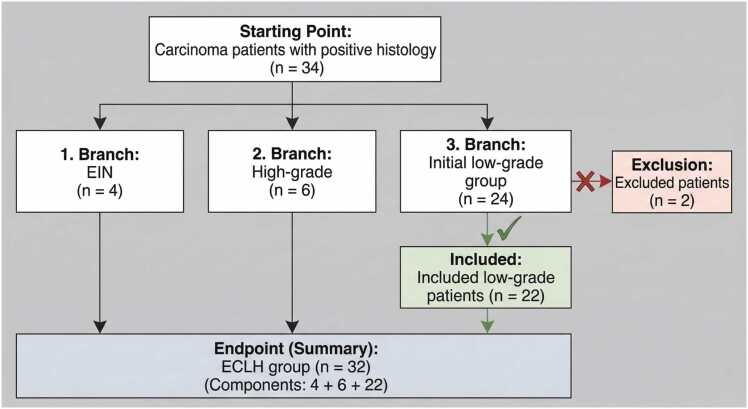
Flow-chart of patient selection for the study.

Based on the description above, we finally differentiated four groups. The first group was composed solely of patients diagnosed with low-grade endometrial cancer (ECL; n = 22). The second group included all patients, encompassing those with endometrial intraepithelial neoplasia, as well as low-grade and high-grade histotype cancers (ECLH; n = 32). The third group consisted of individuals with endometrial intraepithelial neoplasia (EIN; n = 4), the fourth group consisted of the control patients with a benign diagnosis (C; n = 32). (Fig. 1.)

### 3.2 Biophysical, hormonal, and clinical-biochemical parameters

The age and BMI of the endometrial cancer and control groups did not differ significantly (Table 1). There was also no detectable difference when the control group was compared with the ECL group alone or separately with the ECLH or EIN groups. In patient histories, hypertension, type 2 diabetes mellitus (T2DM), and hypothyroidism were the most frequently observed comorbidities. T2DM and hypertension were more prevalent among patients with endometrial carcinoma (T2DM: ECLH 6/32, C 0/32; HT: ECLH 20/32, C 11/32). There were no differences between the groups regarding AST, ALT, GGT, or serum creatinine levels (Table 1). No differences were found between groups on estimated glomerular filtration rate (eGFR), thyroid-stimulating hormone (TSH), serum estradiol (E2), and P4 levels. A higher level of estrone (E1) and E2 was observed in the ECLH group compared to the control. Still, the difference did not reach statistical significance for either hormone (Table 1).

**Table 1:** Biophysical, hormonal, and clinical-biochemical parameters were examined in the study groups (mean ± SEM).

	ECL (n = 22)	ECLH (n = 32)	**EIN** **(n = 4)**	Control (n = 32)
BMI (kg/m2)^ns^	^34.50 ± 1.48^	^34.08 ± 1.20^	^37.05 ± 2.60^	^30.04 ± 1.31^
Age^ns^	^60.45 ± 2.65^	^59.34 ± 2.37^	46.75^± 5.17^	^54.34 ± 1.69^
AST (IU/L)^ns^	^24.72 ± 1.34^	^23.51 ± 1.05^	^20.50 ± 1.708^	^21.56 ± 1.60^
ALT (IU/L)^ns^	^27.45 ± 3.44^	^25.09± 2.59^	^20.75 ± 2.016^	^23.83 ± 2.69^
GGT (IU/L)^ns^	^44.36 ± 10.75^	^37.51 ± 7.91^	^25.00 ± 8.69^	^26.18 ± 4.19^
TSH (mIU/L)^ns^	^2.72 ± 0.49^	^2.50 ± 0.35^	^2.283 ± 0.09^	^1.37 ± 0.36^
Creatinine (μmol/L)^ns^	^72.68 ± 4.29^	^74.58 ± 3.82^	^62.50 ± 3.52^	^73.86 ± 2.32^
eGFR (ml/p/1,73 m2)^ns^	^74.76 ± 3.19^	^73.80 ± 2.94^	^90.00 ± 0.00^	^78.55 ± 2.33^
E2 (pg/ml)^ns^	^38.18 ± 13.87^	^78.87 ± 42.13^	^26.81 ± 5.26^	^40.23 ± 8.98^
P4 (ng/ml)^ns^	^0.64 ± 0.19^	^0.62 ± 0.16^	^0.21 ± 0.08^	^0.78 ± 0.31^
E1(pg/ml)^ns^	^65.30 ± 12.75^	^68.79 ± 11.31^	^67.76 ± 26.39^	^35.39 ± 8.09^

### 3.3 Serum and endometrial tissue glyphosate levels

**Fig. 2:**
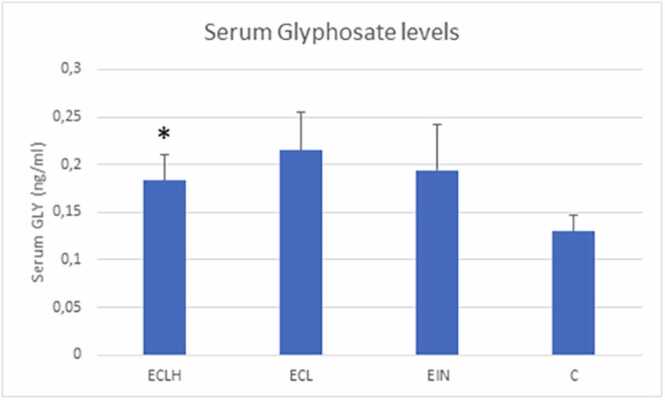
Glyphosate level in serum in study groups (mean±SEM). ECLH: Low-grade and High-grade endometrial cancer (n = 32); ECL: Low-grade endometrial cancer (n = 22); EIN: Endometrial intraepithelial neoplasia (n = 4) and C: control (n = 32). Glyphosateconcentration concentration in serum, measured in ECLH, ECL, EIN and C groups. *ECLH serum level significantly differs from control serum level; GLY serum: ECLH serum 0.1829 ± 0.0272 ng/ml; ECL serum 0.2148 ± 0.0406 ng/ml; EIN serum 0.1938 ± 0.00488 ng/ml and C serum 0.1297 ± 0.0167 ng/ml.

Serum GLY level was significantly higher in the ECLH group than in the control group (p < 0,05). However, it was not significantly higher in the EIN and ECL groups than in the controls. (Fig. 2.)

**Fig. 3:**
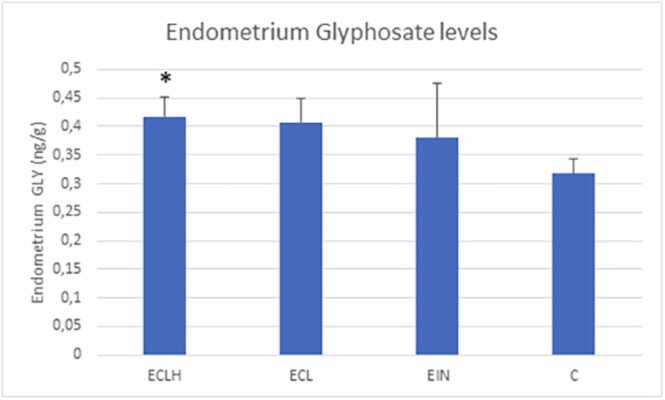
Glyphosate level in endometrial tissue in study groups (mean±SEM). ECLH: Low-grade and High-grade endometrial cancer (n = 32); ECL: Low-grade endometrial cancer (n = 22); EIN: Endometrial intraepithelial neoplasia (n = 4) and C: control (n = 32). GlyphosateconcentrationGLY concentration in endometrial tissue, measured in ECLH, ECL, EIN and C groups. We adjusted the mean concentration values for the endometrium by applying a serum density value of 1.025 g/ml as a conversion factor: from pg/ml to pg/g and ng/ml to ng/g. [18] *ECLH endometrium level shows a significant difference from the control serum level; GLY endometrium: ECLH endometrium 0.4174 ± 0.3461 ng/g; ECL endometrium 0.4073 ± 0.04162 ng/g; EIN endometrium 0.3798 ± 0.09556 ng/g and C endometrium 0.318 ± 0.0251 ng/g.

GLY concentration in the endometrium was significantly higher in the ECLH group compared to the control cases (p < 0.05). However, it was not significantly higher than that of the ECL and control groups or the EIN and control groups. (Fig. 3.)

**Fig. 4:**
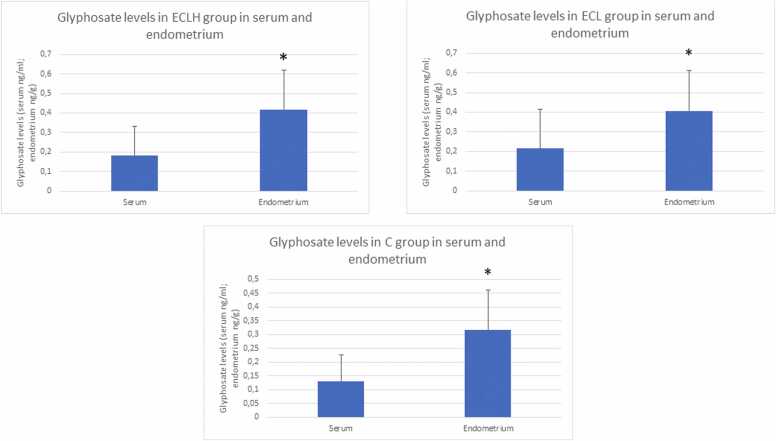
Glyphosate level in serum and endometrial tissue in study groups (mean±SEM). ECLH: Low-grade and High-grade endometrial cancer (n = 32); ECL: Low-grade endometrial cancer (n = 22); and C: control (n = 32). Glyphosateconcentration concentration in endometrial tissue, measured in ECLH, ECL, and C groups. We converted concentration mean values in the case of endometriumendometrium using 1025 g/ml serum density value as a multiplier: pg/ml to pg/g, ng/ml to ng/g. [18] *Endometrium level significantly differs from control serum level in all study groups. P < 0.0001; GLY levels: ECLH serum 0.1829 ± 0.0272 ng/ml; endometrium 0.4174 ± 0.3461 ng/g; ECL serum 0.2148 ± 0.0406 ng/ml; endometrium 0.4073 ± 0.04162 ng/g; and C serum 0.1297 ± 0.0167 ng/ml; endometrium 0.318 ± 0.0251 ng/g.

When analyzing only the high-grade cases, we found significantly higher GLY saturation than in control cases (p < 0.05) in both endometrial tissue and serum. The comparison of serum and endometrium GLY levels was based on a conversion factor that equates 1 ml of serum to 1.025 g [18]. The results revealed that the GLY content in the endometrium was significantly higher than in the serum for all analyzed groups (ECLH; ECL; C) (p < 0.05). (Fig. 4)

When analyzing the subgroups independently, it became evident that the statistical significance initially observed in the combined cancer cohort was driven exclusively by the high-grade cases (n = 6). This specific high-grade subgroup demonstrated significantly higher GLY saturation compared to the control cases (p < 0.05) in both endometrial tissue and serum, whereas the low-grade and EIN groups did not show a statistically significant difference from the controls.

There was a multiple linear regression correlation between serum GLY and E2 levels in all patients (p < 0.05). When we computed correlations among biometric data, hormone levels, and GLY levels in the endometrium, no significant correlations were found in either the patient groups or the control group.

### 3.4 Correlation between endometrial GLY and mycotoxin concentrations

The serum and endometrium tissue concentrations of the most essential mycotoxins and GLY are shown in Table 2 (Table 2).

**Table 2:** GLY and Mycotoxin levels of the diferent groups in the serum and endometrium. Data are per ml for serum levels and per gram for endometrial tissue in mean±SEM.

	**GLY** **(ng/ml – serum; ng/g - endometrium)**	**AFs** **(pg/ml – serum; pg/g - endometrium)**	**DON** **(ng/ml – serum; ng/g - endometrium)**	**OTA** **(pg/ml – serum; pg/g - endometrium)**	**FB1** **(pg/ml – serum; pg/g - endometrium)**	**ZEN** **(pg/ml – serum; pg/g - endometrium)**	**αZOL** **(pg/ml – serum; pg/g - endometrium)**	**T2/HT2** **(ng/ml – serum; ng/g - endometrium)**
**ECL serum** **(n = 22)**	0,2164^±^ 0,04419	18,79^±^ 5603	8504^±^ 1730	9273^±^ 1882	405,5^±^ 121,7	126,8^±^ 34,00	424,3^±^ 86,64	0,8621^±^ 0,09578
**ECL endometrium** **(n = 22)**	0,4083^±^ 0,04547	160,6^±^ 26,02	20,95^±^ 4075	47,98^±^ 10,62	78,73^±^ 17,20	222,3^±^ 40,11	1217^±^ 183,0	2951^±^ 0,4204
**ECLH serum** **(n = 32)**	0,2428^±^ 0,04258	23,18^±^ 6050	9333^±^ 1461	11,94^±^ 2327	401,3^±^ 112,9	206,0^±^ 94,91	484,1^±^ 71,03	0,7672^±^ 0,07435
**ECLH endometrium** **(n = 32)**	0,4187^±^ 0,03679	208,9^±^ 34,43	29,18^±^ 5839	49,75^±^ 8788	85,59^±^ 17,22	189,6^±^ 29,66	1176^±^ 144,9	3334^±^ 0,3431
**EIN serum** **(n = 4)**	0,1938^±^ 0,0488	32,96^±^ 22,69	8223^±^ 3649	18,00^±^ 6831	127,0^±^ 48,23	76,83^±^ 57,71	761,5^±^ 224,5	0,5710^±^ 0,1960
**EIN endometrium** **(n = 4)**	0,3798^±^ 0,09556	125,3^±^ 122,9	52,14^±^ 25,64	25,00^±^ 10,63	33,75^±^ 25,03	53,83^±^ 29,42	1233^±^ 551,1	3633^±^ 0,9424
**Control serum** **(n = 32)**	0,1313^±^ 0,01716	2257^±^ 1024	5419^±^ 1101	7875^±^ 1616	232,4^±^ 62,65	68,59^±^ 12,85	481,2^±^ 82,66	0,9073^±^ 0,09749
**Control endomerium** **(n = 32)**	0,3186^±^ 0,02595	87,24^±^ 26,65	26,87^±^ 4452	55,59^±^ 10,83	27,64^±^ 8905	110,8^±^ 21,08	938,6^±^ 145,5	4555^±^ 0,6479

**Fig. 5:**
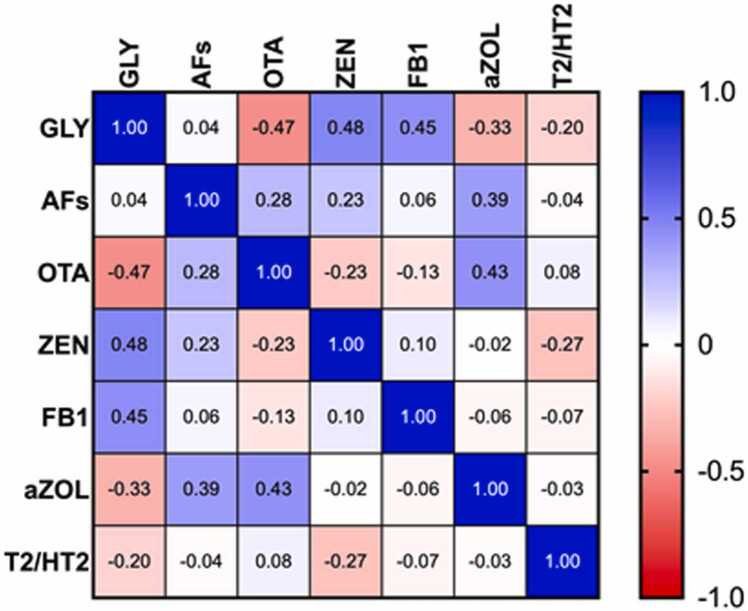
The correlation matrix of endometrial GLY levels. The figure shows the correlations between GLY and mycotoxin levels measured in the endometrium (patients and controls, n = 64). The blue indicates the positive, and the red indicates the negative correlations. The r values are shown in the table.

The level of endometrial GLY showed a significant correlation with the levels of several tested mycotoxins. A strong positive correlation was detected with the endometrial ZEN (p < 0.0001, r = 0.48) and Fumonisin B1 (FB1) levels (p < 0.001, r = 0.45). In contrast, the endometrial ochratoxin-A (OTA, p < 0.0001, r = -0.47) and α-zearalenol (α-ZOL, p < 0.01, r = -0.33) concentrations showed a strong negative correlation. The linear regression correlation between endometrial GLY concentration and endometrial ZEN is particularly noteworthy (p < 0.0001). A linear regression relationship was also demonstrated between the endometrium and serum GLY concentrations (p < 0.0001). (Fig. 5)

## 4 Discussion

With our current study, we aimed to obtain a cross-sectional view of GLY level in endometrial tissue, compare it to serum GLY levels, and determine the possible association of higher level of GLY with uterine cancer formation. Additionally, we also wanted to consider and emphasize interactions of GLY with other relevant, acting endocrine disrupting chemicals/pollutants (EDCs), such as some mycotoxins.

The patient selection process commenced in September 2023 and continued until June 2024. The main objective of the study was to provide a cross-sectional analysis of GLY levels in serum and endometrial tissue among women who had been previously diagnosed with endometrial cancer during perimenopause or postmenopause, as well as among healthy controls.

Age, BMI, T2DM, and hypertension are the most prevalent risk factors for endometrial cancer [12]. There were no notable differences in age or BMI across the groups, making them comparable. Hypertension and diabetes mellitus or prediabetes were the most prevalent comorbidities in the cancer groups.

Regarding median levels of serum E1 and E2 hormone levels, they were higher in cancer patients than in the control group. However, we did not identify the differences significant. The primary sources of estrogen hormones in both menopausal and perimenopausal women are the endometrial tissue, the adrenal gland, and peripheral fat tissue. Since the controls were also matched in BMI, we can assume that there is an overproduction of in situ estrogens in endometrial tissue and/or other estrogen-sensitive tissues [19].

The prognosis, hormone reliance, and responsiveness to treatments of high-grade endometrial cancer differ from those of low-grade tumors. Consequently, a more uniform group was formed, consisting of only low-grade cancer patients (ECL) and a group with all cancer patients (ECLH). Because endometrial intraepithelial neoplasia is a premalignancy with the potential to develop into cancer, patients with this diagnosis were placed in a different group (EIN). However, due to the small number of these cases, the data only showed a tendency regarding GLY levels compared to the cancer and control groups.

In our previous study, we concluded that we examined the same population of patients and controls and were interested in mycotoxin levels in endometrial tissue and serum. Using our earlier results and our stored endometrium and serum samples, we compared GLY and mycotoxin levels. We found a strong correlation between GLY and ZEN levels in endometrial tissues.

The novelty of this study lies in its potential to be the first investigation examining GLY in human endometrial tissue. Our findings might allow us to conclude that GLY may be accumulated in endometrial tissues; however, endometrial GLY levels were significantly higher only in cases of high-grade endometrial cancer, and endometrial levels were significantly greater than serum levels in all examined groups.

Crucially, the cross-sectional design of this study represents a limitation, as measuring GLY and ZEN levels at a single time point during surgery inherently restricts our ability to distinguish cause from consequence. Therefore, it remains undetermined whether the accumulation of these contaminants or maybe relevant residues preceded tumor initiation or occurred subsequently. The associations observed should be interpreted as potential risk factors or co-factors in a complex microenvironment rather than direct drivers of pathogenesis. In our opinion further limitation is the low number of involved cases, by which we can only see tendencies.

We found no prior studies suggesting GLY levels and/or deposition in human tissues, as a significant portion of GLY is primarily eliminated from the body unchanged and unmetabolized [20]. We selected patients of similar age as controls, among whom endometrial atrophy was identified in most cases. However, dedicated scientific or official surveys or background research were not applied; based on our questions and anamnesis, the patients showed “normal life” without (knowing of) an extra potential chance to reach accidental exposure to GLY or other GBHs. In postmenopausal women, the absence of cyclic changes may lead to variations in the accumulation rate of certain mycotoxins at the tissue level due to shifts in metabolic processes, hormonal conditions, and circulatory factors, potentially prolonging elimination.

Therefore, our results might provide a reasonable basis for searching for a logical explanation for our findings, which can be further examined in the future. It is known that GLY has a structure similar to glycine, a vital amino acid component of collagens, the most abundant proteins in animals [21], [22]. Collagen consists of a triple helix formed from three polypeptide chains, with glycine, proline, and hydroxyproline making up 57% of their total amino acids [21], [23]. Interestingly, while GLY is intended to target plant enzymes, studies indicate that it may have unintended effects on animals and humans. For example, GLY can be absorbed through epithelial tissues, including the gastrointestinal and respiratory tracts, which could lead to systemic effects [24].

Additionally, exposure to GLY has been linked to alterations in gut microbiome composition and functionality, which may impact human health [25]. The structural similarity between GLY and glycine raises concerns about possible interference with collagen synthesis and/or other biological processes. Although GLY is regarded as having low toxicity to humans, its widespread use and potential for environmental accumulation necessitate further investigation into its long-term effects on animal and human health, particularly concerning collagen-related processes [21], [22], [25].

Due to the lack of in vivo studies, no data regarding the normal fertile endometrium is available. Compared to the controls, a significantly higher serum GLY level was measured in the ECLH group.

A critical distinction in our findings is that significantly elevated GLY levels were specifically associated with high-grade endometrial cancer cases, rather than the broader category of endometrial malignancies. However, it is essential to acknowledge the small sample size of this high-grade subgroup (n = 6). Consequently, while the difference is statistically significant, these observations must be interpreted cautiously as preliminary findings. This specific association highlights the need for rigorous validation in larger, independent cohorts to confirm whether GLY accumulation is distinctly linked to tumor grade and aggressiveness.

Although GLY and its metabolites have not been proven as direct etiological cofactors of many cancers, including those of the kidney, bladder, colon, and rectum, GLY might contribute to hormone-dependent cancers as estrogen-sensitizing agents [26]. The potential role of GLY in breast cancer is controversial [27]. However, GLY and its primary metabolite - AMPA, exhibit estrogenic effects by interacting with ESRs, particularly ESR1, and alternative signaling pathways [28]. GLY has been shown to stimulate cell proliferation in ESR1-positive cells via non-genomic estrogen–receptor/extracellular signal-regulated kinase 1/2 (ER/ERK1/2) pathways, suggesting AMPA may have similar effects [29]. GLY promotes breast cancer cell growth through ESRs. In cholangiocarcinoma cells expressing ESR1, GLY-induced proliferation via the ER/ERK1/2 pathway, an effect inhibited by ESR antagonists and mitogen-activated protein kinase (MEK) inhibitors, indicating both ESR1 and ESR2 and alternative pathways are involved.

One limitation of the study is that we measured only glyphosate in the endometrial tissue and not aminomethylphosphonic acid (AMPA), which is the main biotransformation metabolite and environmental degradation product of glyphosate. The presence and quantity of AMPA could provide further information about the full spectrum of glyphosate exposure, as the biological effects and elimination patterns of the metabolite may differ from those of the original herbicide [20]. AMPA also possesses endocrine-disrupting chemical (EDC) properties, as it is capable of affecting the functioning of the hormonal system and thus potentially interfering with endocrine regulation [30].

A significant correlation has been observed between higher levels of ZEN and the presence of endometrial cancer [15]. The estrogenic activity of ZEN and α-ZOL has been demonstrated in various studies, with α-ZOL showing a higher affinity for ERs and inducing a stronger proliferative effect in estrogen-dependent cells compared to ZEN [31], [32]. In the absence of literature data regarding the negative correlation between glyphosate and αZOL concentrations, it appears that this is due to either competition for 3α-hydroxysteroid dehydrogenase binding sites or entry into cells via a common active mechanism.

ZEN exerts estrogenic effects primarily through ESR1 but also activates alternative pathways such as G protein-coupled receptor 30 [33], [34]. G protein-coupled receptor 30 in pig pituitary cells, leading to protein kinase cascades. Phosphorylation of protein kinase C (PKC), ERK, and p38 mitogen-activated protein kinase (p38MAPK), ESR2 partially counteracts ZEN-induced oxidative stress. Lim-homebox 3 (LHX3) is a potential mediator of the effects of ZEN on gonadotropes in the pig pituitary [34], [35]. ZEN activates multiple estrogen signaling pathways, contributing to its complex biological effects and highlighting the importance of considering various pathways in studying its impact on reproductive and endocrine systems. Understanding these mechanisms is crucial, as GLY and AMPA are widespread contaminants that affect reproductive health [36]. When we consider that endometrial GLY levels and elevated endometrial ZEN levels showed a significant correlation, we can infer that these agents may have a synergistic effect in estrogen-driven diseases, such as uterine cancer.

It's increasingly important to consider the subtle yet significant role that even very low levels of environmental contaminants, such as multimycotoxins and pesticides like GLY, may play a role in cancer development, particularly in hormone-dependent malignancies: Both glyphosate and certain mycotoxins, such as ZEN, are recognized as EDCs. This means they can mimic or interfere with the body's natural hormones, particularly estrogens. In hormone-sensitive tissues, such as the endometrium, this can disrupt normal cellular processes, potentially driving abnormal growth and increasing the risk of cancer. GLY has been shown to activate estrogen receptors (ESRs), while ZEN is a known xenoestrogen that binds to ESRs and other signaling pathways.

Synergistic and co-amplification effects: Perhaps most importantly, these contaminants rarely act in isolation. The presence of multiple mycotoxins and pesticides can lead to synergistic or co-amplification effects. For instance, a strong positive correlation has been observed between endometrial GLY levels and estrogenic mycotoxins, such as ZEN. This suggests that even low-level exposure to a combination of these agents could collectively exacerbate their detrimental impact on hormone-driven diseases, potentially accelerating tumor development. The complex interplay of these substances may exert a greater pathological effect than each compound alone.

To contextualize these findings within the study’s geographic region, it is essential to acknowledge that both GLY and ZEN are prevalent environmental contaminants in Central Europe. Fungi of the Fusarium genus, which produce ZEN, are highly prevalent in the European temperate climate and frequently contaminate cereals and feed [37]. Recent assessments by the European Environment Agency indicate that while approximately 25% of crops exceed EU regulatory limits for mycotoxins, actual contamination occurs at detectable levels in 60–80% of crops [38]. A specific survey of grain-based swine feed in Hungary revealed that ZEN was present above the limit of detection in 100% of the tested samples, indicating a persistent risk of entry into the food chain [37]. ZEN is a recognized xenoestrogen and endocrine disruptor that can cause reproductive issues in humans and animals [39]. Furthermore, recent overviews of European human biomonitoring studies have highlighted widespread exposure to GLY among the general EU population [40]. Agricultural soil analyses have also demonstrated that pesticide residues, including glyphosate (GLY), are frequently retained in European cultivated soils [41]. Consequently, the cumulative risk assessment of chemical mixtures in food is gaining significant attention, as individual compounds that do not exceed safety limits may collectively pose health risks owing to their additive or synergistic effects on specific organ systems [42]. These regional prevalence data robustly support the clinical relevance of our findings, demonstrating that chronic low-dose co-exposure to GLY and ZEN is a realistic environmental hazard for the investigated patient population.

Implications for Hormone-Dependent Cancers: For hormone-dependent cancers, such as endometrial cancer, breast cancer, and prostate cancer, understanding these environmental factors becomes paramount. While direct causal links require further extensive research, the evidence suggests that these low-level, combined exposures may be potential contributors to the pathogenesis. They may "prime" the environment for cancer development or progression by influencing hormonal pathways and cellular signaling.

## 5 Conclusions

This study demonstrates the elevated level of GLY in human endometrial tissue, especially in patients with high-grade endometrial cancer. The notable correlation between GLY levels and the estrogenic mycotoxin ZEN may suggest a potential synergistic effect in hormone-dependent malignancies. Although the exact mechanisms remain unclear, the findings raise significant concerns about the possible role of GLY as an EDC contributing to the pathogenesis of uterine cancer. While the acute toxic effects of GLY and AMPA on mammals are minimal, some animal studies suggest potential health effects linked to chronic, ultra-low doses due to the accumulation of these compounds in the environment. Given the widespread use of GBHs and their detection in human biological samples, further research is essential to establish causal relationships and evaluate long-term health risks. Future studies should aim to clarify the molecular interactions between GLY, hormonal pathways, and carcinogenic processes, as well as investigate potential regulatory thresholds to reduce exposure risks. Our study also highlights inconsistencies in the literature concerning the effects of GLY and emphasizes the need for further research, particularly on low-dose, long-term exposure to GLY. To clarify the problem mentioned above and confirm our results, developing a more sensitive HPLC method for measuring glyphosate content in the endometrium would be necessary.

In conclusion, clinicians must be aware that the toxicological landscape is evolving. It is not just about high-dose acute exposures but increasingly about the chronic, low-level presence and interplay of various environmental contaminants. Recognizing the potential for multi-mycotoxins and pesticides to accumulate in specific tissues and exert synergistic endocrine-disrupting effects is vital for a holistic approach to cancer prevention, risk assessment, and ultimately, improving patient outcomes. Further research into the molecular mechanisms and long-term consequences of these complex exposures is imperative for guiding future clinical practice and public health recommendations.

## CRediT authorship contribution statement

**Apolka Szentirmay:** Writing – review & editing. **Györgyi Fekécs:** Writing – review & editing. **Patrik Plank:** Investigation. **Zsófia Molnár:** Project administration, Investigation. **Levente Sára:** Writing – original draft, Validation, Supervision, Resources, Project administration, Methodology, Investigation, Funding acquisition, Formal analysis, Data curation, Conceptualization. **Zsuzsanna Szőke:** Writing – original draft, Validation, Supervision, Resources, Project administration, Methodology, Investigation, Funding acquisition, Formal analysis, Conceptualization. **Márkó Unicsovics:** Writing – original draft, Visualization, Project administration, Investigation, Formal analysis, Data curation, Conceptualization. **György Nagyéri:** Investigation. **Katalin Posta:** Writing – review & editing, Resources, Funding acquisition. **Nándor Ács:** Writing – review & editing. **Szabolcs Várbíró:** Methodology.

## Funding acquisition

Levente Sara; Zsuzsanna Szoke; Katalin Posta

## Funding

This research was supported by the Hungarian National Research, Development and Innovation Office, grant number NKFI K 132687, the Flagship Research Groups Programme of the Hungarian University of Agriculture and Life Sciences, and the Hungarian National Labora-tory Project, grant number RRF-2.3.1-21-2022-00007, Agribiotechnology and Precision Breeding for Food Security National Laboratory.

## Declaration of Competing Interest

The authors declare that they have no known competing financial interests or personal relationships that could have appeared to influence the work reported in this paper.

## Data Availability

Data will be made available on request.
